# Otorhinolaryngological myiasis: the problem and its presentations in the weak and forgotten

**DOI:** 10.4314/gmj.v54i3.8

**Published:** 2020-09

**Authors:** Amit K Rana, Rohit Sharma, Vinit K Sharma, Ashish Mehrotra, Rachana Singh

**Affiliations:** 1 Departments of Otorhinolaryngology and Head and Neck Surgery, SRMS Institute of Medical Sciences, Bareilly (UP), India; 2 Apollo Indraprastha Hospital, New Delhi, India

**Keywords:** Myiasis, Ivermectin, screwworm, *Chrysomya bezziana*, *Musca domestica*, *Lucilia sericata*

## Abstract

**Introduction:**

Myiasis is common in tropical regions, but now increasing incidence is seen in the west due to international travel. Otorhinolaryngological myiasis is uncommon and is seen in diabetics, alcoholics or patients unable in self-care.

**Objectives:**

To study presentations of otorhinolaryngological myiasis, identify associated risk factors and species of flies causing myiasis.

**Methods:**

Clinical findings and co-morbidities of 67 myiasis cases were noted. Maggots were identified, manually removed, and patients were managed with topical treatment, systemic ivermectin and antibiotics.

**Findings:**

Thirty-three nasal myiasis, 13 aural myiasis and 5 patients with oral myiasis were noted. Seven patients with head neck wounds myiasis and nine patients of tracheostome myiasis were recorded.

**Discussion:**

Warm humid climate of tropical regions is a major concern along with co-existing conditions like poor sanitation, alcoholism, psychiatric diseases and neuropathies. Hesitancy is seen in attendants and health care professionals to deal with myiasis.

**Conclusion:**

Awareness about risk factors is important in avoiding myiasis along with prompt treatment which reduces morbidity. Tracheostome myiasis is an under-documented entity rather than a rare presentation.

**Funding:**

None

## Introduction

Myiasis is an infestation of parasitic dipterous larvae in living human and vertebral animals. The term myiasis was coined by Rev FW Hope in 1840^1^ and comes from the Greek word “muia” or “mya” meaning fly and “iasis” meaning disease. The most common flies causing human myiasis are of the order Diptera, family Calliphoridae, species calyptratae - *Chrysomya bezziana* (named to honour Italian entomologist *Mario Bezzi)* seen in South Asia, India, Africa, Saudi Arabia, Indonesia, Guinea and the Persian Gulf.

Myiasis is a well-known condition on the Indian sub-continent, found in sores and wounds of animals. Human infection with *C. bezziana* was first reported by Abed Benamara et al. from Algeria in 1997.^2^ Authors have also reported Drosophillidea causing myiasis in the nose and eyes.^3^ The dipterous larvae feed on the host tissue and body fluids to grow and complete their life cycle.

Larvae of *M. domestica* (Indian housefly) are seen commonly in wounds. Bishopp was the first one to classify myiasis according to their location on host.^4^ Myiasis infection can be wound myiasis, cavitary myiasis, sanguinivorous or even migratory myiasis. Larvae may be obligate parasites affecting undamaged skin, semi-specific where the larvae are laid on damaged skin usually associated with wounds or facultative parasites which can accidentally infect humans.

Human myiasis has a worldwide distribution, but its presence in the western world is extremely rare. It is an uncommon condition and is seen more in tropical and subtropical areas. Even though uncommon in the western world, it is on the rise in non-endemic regions with increased international travel. It is the fourth most common travel-associated skin disease, representing up to 7.3% to 11% of cases.

This has made physicians more aware of myiasis in places where the infection is not common.^5^, ^6^, ^7^

Myiasis stays largely unknown as epidemiological data on otorhinolaryngological myiasis is very sparse, with just a handful of studies, mostly case reports with incomplete registries due to a lack of follow up with patients. The maggots are often destroyed as soon as they are extracted and identification of species responsible is rarely attempted. Also, entomologists are not easily accessible in developing countries where this is an endemic problem.

Although myiasis is seen in every part of the body, the scope of this study is to record manifestations of myiasis in otorhinolaryngological practice. In our practice, we most commonly encounter cavitary myiasis, and so the infection is named after the organ or cavity infested like nasal myiasis, aural myiasis, tracheostome myiasis, and oral myiasis. No studies have been conducted for the condition in this region to establish epidemiology, associated risk factors and presentations of human otorhinolaryngological myiasis. Our study aims to fill this gap and provide a better understanding of this health problem in North India. The study looked at the presentation of otorhinolaryngological myiasis, identify associated risk factors and the species of flies causing myiasis.

## Methods

This observational study was conducted in the Department of Otorhinolaryngology and Head Neck Surgery of a tertiary care centre in Bareilly (UP) India. The time duration of the study was July 2014 to July 2018. Sixtyseven cases of myiasis presented to OPD were evaluated in this study. This study was done after due permission from the Institutional Ethics and Research Committee vide letter no ECC/2014/119 dated 26.06.2014. Consent of patients/family was taken to use the information and pictures of patients in the study.

A detailed history of the symptoms was recorded, along with any significant travel history, history of co-morbidities like diabetes, long- term debilitating disease, alcoholism, mental status and malignancy. After a complete examination, any visible maggots were removed mechanically either with headlight or endoscope. Patients were managed with antibiotics and anti-inflammatory drugs to treat any secondary infection due to tissue destruction. Maggot extraction was repeated till no more maggots were extracted for 48 hours, followed by an endoscopic examination to confirm the completion of treatment. After removal, the larvae were killed by placing them immediately into hot water for 30 seconds.

They were then transferred to a 70% isopropyl alcohol solution to preserve their natural color and prevent decay. Storage in formalin was avoided as it hardens the tissue making identification of species impossible. In the absence of a trained entomological expert, maggots were examined for features of identification to establish species with online reference to the “Natural History of Museum^8^ (http://www.nhm.ac.uk/research-curation/scientific-resources/taxonomy-systematics/myiasis-larvae/index.html)” key to myiasis causing larvae. Larvae were also identified by its morphological features using a key and description given in a monograph of polish Calliphoridae.^9^ Cross verification of species was done with the help of faculty of the Department of Community Medicine of the institution.

## Results

Myiasis was seen to present equally in both sexes, with greater occurrence in those over 50 years of age. Mean age was 62.57 years in males and 61.85 years in females (Table 1).

**Table 1:** Age and gender distribution of patients

Age (yrs)	Nasal myiasis		Aural myiasis		Tracheostome Myiasis		Head neck Wound myiasis		Oral Myiasis		Total		
	M	F	M	F	M	F	M	F	M	F	M	F	
**0–10**	-	2	3	-	-	-	-	1	-	-	3	3	06(08.95%)
**11–20**	-	-	-	1	-	-	-	-	-	-	-	1	01(01.49%)
**21–30**	-	-	-	-	-	-	-	2	-	-	-	2	02(2.98%)
**31–40**	-	-	-	-	-	-	-	-	-	-	-	-	-
**41–50**	1	4	-	-	-	-	-	-	-	-	1	4	05(07.46%)
**51–60**	5	6	3	-	-	-	-	-	-	-	8	6	14(20.89%)
**61–70**	4	7	2	4	5	1	2	-	3	1	16	13	29(43.28%)
**>70**	1	3	-	-	3	-	-	-	2	1	6	4	10(14.92%)
**Total**	11	22	8	5	8	1	2	3	5	2	34	33	67(100.00%)

Diabetes (62.68%) emerged as the most common associated finding and possibly an important risk factor in patients with myiasis. Patients who lived alone or were homeless (52.23%), non-ambulatory (32.83%) and suffered from alcoholism (22.38%) were seen to be more vulnerable to the infestation of maggots. Patients with psychiatric disorders (14.92%) were also more likely to suffer from myiasis (Table 2).

**Table 2:** Associated risk factors

Risk Factor	Nasal myiasis		Aural myiasis		Tracheostome Myiasis		Head neck Wound myiasis		Oral Myiasis		Total
	M	F	M	F	M	F	M	F	M	F	
**Diabetes**	7	14	4	2	3	1	2	2	3	2	42(62.68%)
**Mental Disorders**	2	5	-	-	-	-	1	-	2	-	10(14.92%)
**Alcoholism**	8	3	2	-	-	-	1	-	1	-	15(22.38%)
**Non ambulatory**	2	8	3	4	2	-	1	-	2	-	22(32.83%)
**Homeless**	6	12	5	2	4	1	1		2	2	35(52.23%)
**Malignancy**		2	1		6	1	2	2			14(20.89%)
**Atrophic rhinitis**	2	3	1	-	-	-	-	-	-	-	06(8.95%)
**Leprosy**	1	1		-	-	-	-	-	-	-	02(2.98%)

In our study, 33 patients were seen to be suffering from nasal myiasis. One patient presented with orbital maggots but was later found to have originated as a case of nasal myiasis where maggots breached the medial orbital bone and entered orbit, causing the complete destruction of the left eyeball. This patient was managed and then transferred to the Ophthalmology Department for further management (Table 1)

**Figure 1:**
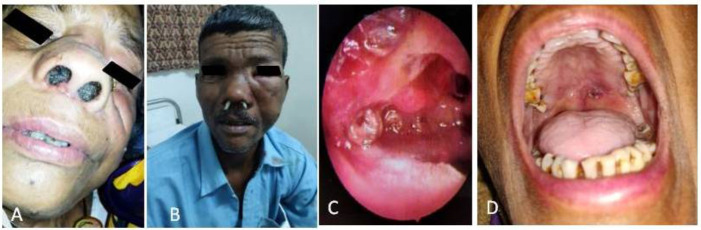
Nasal myasis (A patient with nose filled with maggots (B) Turpentine wick inside nose (C) Nasal endoscopy showing maggots (D) Impending palatal perforation

Aural myiasis was seen involving the external ear and middle ear in 13 patients with larvae protruding out of ear canal. It was seen more in small children or in old diabetics with sensory loss. On exposure to light, these larvae retreated to the middle ear and soft tissue tunnels they had created. Bleeding was present in 10(76.92%) patients. Twelve patients (92.30%) reported severe pain and tympanic membrane perforation was present in all 13 (100.00%) cases. One case of bilateral disease was also reported. Ossicular destruction resulting in hearing loss was seen in 4(30.76%) patients (Table 1 and Figure 1).

Primary treatment was the application of a toxic substance to the larva, production of localized hypoxia to force the emergence of the larva and mechanical or surgical removal of maggots.

In nasal myiasis, 4% Xylocaine and turpentine oil mixture were instilled in the form of drops or soaked cotton wick. Nasal douching with a lukewarm solution of Sodium chloride, Sodium bicarbonate and Sodium borate in the ratio of 2:1:1 was then utilized for extraction. Repeat endoscopic removal was done to ensure completely removed of larvae. In cases of aural myiasis, the external auditory canal was irrigated with 4% Xylocaine to restrict movements of larvae. Larvae were then removed under microscopic guidance. Repetitive cleaning of the canal and middle ear was done with normal saline with instillation of antibiotic ear drops containing steroid to reduce local inflammation and infection.

**Figure 2:**
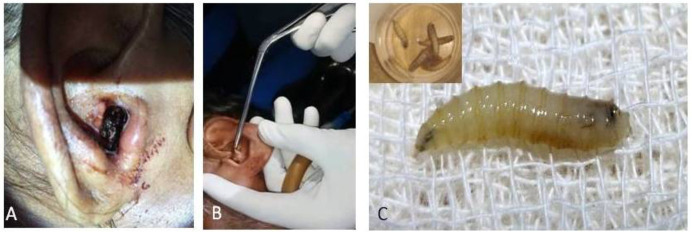
Aural myasis (A) Maggots visible in external auditory canal (B) Manual extraction of maggots (C) Maggots for identification (Insert – extracted maggots)

A *statim* oral dose of ivermectin (200 µg /kg body weight) at start of treatment was given to the patient. It was found to help reduce the time of stay at hospital significantly (Table 3 and Figure 2).

**Table 3:** Treatment modalities and the comparative effect

Treatment	Mean time taken for clearance of maggots in days			
	Nasal myiasis	Aural myiasis	Tracheostome	Oral/wound
**Manual Extraction only (28 cases)**	9.6±1.6	8.3 ±2.3	6.7 ±0.8	4.4 ±1.5
**Manual extraction Plus ivermectin (39** **cases)**	6.4 ±1.3	5.2 ±1.4	4.9 ±1.2	3.8 ±1.1

On identification, it was observed that of 41 larvae samples which could be collected, examined and identified by the keys stated above, 26(63.41%) belonged to *Chrysomya bezziana*. Larvae of *Musca domestica* were identified in 14(34.14%) cases. One patient who came from Poland had an infestation of *Lucilia sericata* in his ear. *M. domestica* was most commonly seen in cases of oral and wound associated myiasis whereas in other cases *C. bezziana* was prevalent (Table 4).

**Table 4:** Identification of species

Species	Nasal	Aural	Tracheostome	Oral	Wound	Total
***C. Bezziana***	10	9	4	2	1	26(63.41%)
***M. Domestica***	2	3	2	3	4	14(34.14%)
***L. Sericata***	-	1	-	-	-	01(2.43%)

## Discussion

A certain degree of hesitancy is seen in attendants and health care professionals to deal with myiasis. Low socioeconomic status, poor hygiene, alcoholism, psychiatric diseases and neuropathies are frequent co-factors. Flies get attracted to bad odour and lay their eggs on warm, moist surfaces.

The species *C. bezziana* usually lay around 150–200 eggs and larvae come out in around a day. After hatching, larvae bury deep into tissues with help of their sharp hooks and get anchored by the intersegmental spines, scrapping away tissue and damaging small blood vessels.

Tissue destruction may occur by the production of collagenase and proteolytic enzymes.^11^ Haemorrhage may be severe and causes tissue oedema. They also release toxin causing infection and necrosis.^12^

Patients usually present with foreign body sensation, epistaxis, nasal obstruction, rhinorrhea, otorrhea, facial pain and swelling. A characteristic pungent bad odour is also present. Patients often seek treatment at a late stage, when the maggots are either seen crawling out of orifices of the body or cause intense discomfort, pain or bleeding. In otorhinolaryngological practice, such cases sometimes present as an emergency, with life-threatening complications such as airway obstruction. Maggots of C*. bezziana* have a peculiar feature of causing tissue invasion without the need of pre-existing necrosis. They are called ‘old world screwworm’ as they embed themselves into the flesh of host in a screw-like fashion, as deep as 15 cm.^8^ This feature is responsible for septal perforation and destruction of turbinates and even lamina papyracea in nasal myiasis.

In nasal myiasis, the maggots sometimes reach up to the orbit causing blindness, spread medially and cause septal perforations, spread inferiorly and perforate the palate and move to sinuses even reaching the central nervous system.^13^ Arora S et al. in their study reported that 70–75% of cases of ENT myiasis were nasal cases but in our study, we concluded that 33(49.25%) cases present with nasal myiasis.^14^ Mortality rate of aural myiasis with nasal myiasis is 8%.^8^ Proper cleaning of debris with nasal endoscopy reduces the load of myiasis and gives early control.^15^

In our study, 13(19.40%) patients presented with aural myiasis. In a study of 94 pediatric cases conducted by I. Singh et al., 86.14% cases were of aural myiasis.^16^ Aural myiasis can have a range of clinical presentations, including itching in the ear, otalgia, otorrhea, perforation, bleeding, tinnitus, vertigo and restlessness. Multiple manual extractions needed to clear off the larvae load from middle ear cleft.^17^,^18^

**Figure 4:**
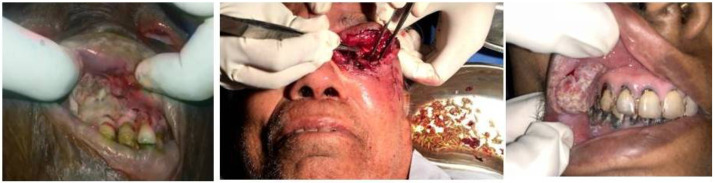
Oral and Head Neck myasis (A) Maggots in oral cavity (B) Involvement of orbit secondary to nasal mysis with extracted maggots (C) Maggots around malignant growth

Infestation of the oral cavity was likely through contaminated food, as the most common areas involved were gingivobuccal sulcus and retromolar trigone. Extensive tissue destruction was seen, and palatal perforation was seen in some cases. Turpentine oil was used locally, and ivermectin was given orally to such patients resulting in a good response. 19,20,21,22 (Figure 4)

**Figure 3:**
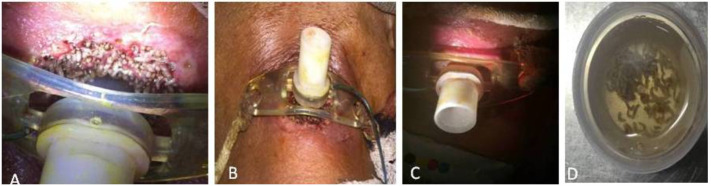
Tracheostome myasis (A) Visible maggots around tracheostome (B) Cleaning with turpentine and manual removal (C) Healed wound after one week (D) Extracted maggots

Tracheostome myiasis is not common, with only a few reported cases in the medical literature. We came across nine cases in 4 years which suggests that in Indian scenario it is not so uncommon, rather it is an under reported entity. It presents with crawling maggots seen between tube and skin. Special care should be taken to avoid removal of the inner tube as there is a risk of larvae falling in the trachea and cause pneumonia. Persistent vegetative state, poor hygiene around the tube, rural setting and odour of mildly infected tracheostome can be factors that predispose patients to this type of myiasis.^1^,^23^,^24^,^25^ (Figure 3)

Malignant ulcers have been implicated as potential sites of myiasis, and such cases have recently been highlighted with better follow up of patients who were previously left to die on their own at the terminal stages. Lack of adequate care and long term non-ambulatory patients with continuous exposure to unhygienic surroundings are predisposed to such infestations. Chigusa et al. suggested that psychiatric and elderly patients with reduced sensations due to which flies lay eggs in their cavities without much opposition are at higher risk .^26^

Posterior spiracles of larvae protrude out of lesion to aid breathing. Occluding the spiracles with oil stops oxygen supply, forcing the larvae to surface for air and hence can be removed with ease. Ivermectin, a semi-synthetic macrolide activates the release of gamma-aminobutyric acid, causing larval death.^21^ Antibiotics are given to treat secondary infections. Scrupulous mechanical removal of the maggots with naked eyes or with the help of endoscope stays the mainstay of treatment. Endoscopy is preferred as it reaches inaccessible areas.^27^

In most cases, it is easy to diagnose myiasis as maggots are visible. The challenge is in patients suffering from cavitary or migratory myiasis and also when the physician is not familiar with this condition. It is important to note a history of travel to endemic regions, climatic conditions and any underlying high-risk patient condition for myiasis. Based on the history, clinical suspicion and analysis in such patients, a presumptive diagnosis can be made and timely treatment given to the patients to avoid complications. We have not seen much literature with *M. domestica* causing tracheostome myiasis, so the presentation of two patients with larvae of this fly was an important finding.

## Conclusion

Poor sanitation is the primary risk factor for human myiasis. Awareness of risk factors and early recognition of warning signs with help of risk factors should be raised. Proper care of old, vegetative, terminally ill and homeless people is paramount. The treatment of myiasis is simple and patients recover well once the correct diagnosis is made and treatment started.

Although the disease is rarely lethal, it can have lifechanging impacts. Education and awareness can help in early recognition and timely intervention, reducing the morbidity of complications. Studies are required to identify the uncommon species responsible for myiasis and their endemic areas so that preventive measures can be taken while travelling in such areas.
